# *Aeromonas* and Human Health Disorders: Clinical Approaches

**DOI:** 10.3389/fmicb.2022.868890

**Published:** 2022-05-31

**Authors:** Rafael Bastos Gonçalves Pessoa, Weslley Felix de Oliveira, Maria Tereza dos Santos Correia, Adriana Fontes, Luana Cassandra Breitenbach Barroso Coelho

**Affiliations:** ^1^Departamento de Bioquímica, Centro de Biociências, Universidade Federal de Pernambuco, Recife, Brazil; ^2^Departamento de Biofísica e Radiobiologia, Centro de Biociências, Universidade Federal de Pernambuco, Recife, Brazil

**Keywords:** microbiology, bacterial infection, human pathogen, prognosis, antibiotic responses

## Abstract

The genus *Aeromonas* comprises more than 30 Gram-negative bacterial species and naturally inhabitants from aquatic environments. These microorganisms, commonly regarded as pathogens of fish and several other animals, have been gaining prominence on medical trial due to its ability to colonize and infect human beings. Besides water, *Aeromonas* are widely spreaded on most varied sources like soil, vegetables, and food; Although its opportunistic nature, they are able to cause infections on immunocompromised or immunocompetent patients. *Aeromonas* species regarded as potential human pathogens are usually *A. hydrophila*, *A. caviae*, and *A. veronii* biovar *sobria*. The main clinical manifestations are gastrointestinal tract disorders, wound, and soft tissue infections, as well as septicemia. Regarding to antibiotic responses, the bacteria present a diversified susceptibility profile and show inherence resistance to ampicillin. *Aeromonas*, as an ascending genus in microbiology, has been carefully studied aiming comprehension and development of methods for detection and medical intervention of infectious processes, not fully elucidated in medicine. This review focuses on current clinical knowledge related to human health disorders caused by *Aeromonas* to contribute on development of efficient approaches able to recognize and impair the pathological processes.

## Introduction

When thinking about *Aeromonas*, the first thing that comes to our minds might be “water” or “fish.” Indeed, bacteria from this genus naturally inhabit aquatic environments and are known as a threat for aquaculture systems (Barger et al., 2021). The hazardousness of Aeromonads for several fish species as well as other waterborne animals has been strongly discussed (Beaz-Hidalgo and Figueras, 2013; Dallaire-Dufresne et al., 2014; Fernández-Bravo and Figueras, 2020). However, the potential of these microorganisms to cause human health disorders must be highlighted.

Worldwide distributed, *Aeromonas* were already isolated from a broad range of sources like fresh water, sewage, soil, fruits, vegetables, and processed food (McMahon and Wilson, 2001; Gonçalves Pessoa et al., 2019; Barger et al., 2021). Thus, contact between these bacteria and human beings can be easily established. Aeromonads are considered emergent pathogens and its detection in various diarrheal stool samples clearly shows they are not as far from clinical routine as other enteric microorganisms (Li et al., 2015; Mohan et al., 2017; Mbuthia et al., 2018). Consequently, interest about the genus *Aeromonas*, which embraces 36 recognized species, has risen over the past years (Fernández-Bravo and Figueras, 2020; Ahmed et al., 2021; Conte et al., 2021).

Several case reports involving Aeromonads infections brought up the diversity of clinical manifestations that these bacteria can provoke to human health. Symptoms range from acute self-limiting diarrhea to lethal sepsis; however, wounds, skin, bones, heart, lungs, eyes, and other organs can be potentially affected (Parker and Shaw, 2011; Ahmed et al., 2021; Meng et al., 2021). Most reports classify *Aeromonas*’s human infections as events caused by “rare” or “uncommon” microorganisms (Lai et al., 2007; Khalil et al., 2013; Hasan et al., 2018; Salehi et al., 2019). Indeed, other bacterial genus causing the same clinical symptoms are more frequent at hospital level. For example, *Campylobacter* spp., *Salmonella* spp., *Shigella* spp., and *Escherichia coli* are listed as the main pathogens in clinical cases of acute gastroenteritis (Fleckenstein et al., 2021). Moreover, *Staphylococcus aureus* and *Streptococcus pyogenes* are frequently detected in skin and wound infections reports (Cefalu et al., 2017; Clebak and Malone, 2018).

Noteworthy, one of the peculiar characteristics of the *Aeromonas* genus is the unreliable phenotypical identification by conventional biochemical tests or commercial systems, like Vitek, API20, and Microscan, which are commonly used in the quotidian of most hospital laboratories (Lamy et al., 2010; Jin et al., 2011; Gonçalves Pessoa et al., 2019; Meng et al., 2021). It is known that *Aeromonas* strains show similarities to other bacterial genera, and accurate data still lies on molecular biology techniques aiming amplification of housekeeping genes for phylogenetic differentiation (Yáñez et al., 2003; Beaz-Hidalgo and Figueras, 2013; Hoel et al., 2017).

The *Aeromonas* possess wide spectra of antibiotic resistance profile and occurrence of multi-resistant strains have already been reported (Galler et al., 2018; Conte et al., 2021). As inhabitants of aquatic environments, these microorganisms can be used as ecological indicators of water pollution since they harbor antibiotic resistance genes obtained from wastewater effluents (Baron et al., 2017; Grilo et al., 2020). Furthermore, the global indiscriminate use of antimicrobials has changed the perspective of medicine regarding bacterial infections for the next years. The incidence of multidrug-resistant microorganisms has increased and it is expected to be the main public health problem in the future (Dimopoulos et al., 2016). Aeromonads are prone to be susceptible to fluoroquinolones but usually resistant to ampicillin and other beta-lactams (Parker and Shaw, 2011; Fernández-Bravo and Figueras, 2020). In a clinical scenario, however, the execution of an antibiogram test must not be dispensed so as to ensure suitable antibiotic prescriptions (Abbott et al., 2003).

In this review, information about the main clinical features of *Aeromonas* infections will be provided to highlight how dangerous these bacteria can be for human beings. The knowledge about epidemiology, differential diagnosis, and strategies for antibiotic therapy are also explored.

## *Aeromonas* Epidemiological Profile

*Aeromonas* species generally associated with human infections include *A. caviae*, *A. schubertii*, *A. hydrophila*, *A. veronii* (biovars *veronii* and *sobria*), and *A. jandaei* (Lamy et al., 2010). These microorganisms have been isolated in hospitals worldwide, and different types of clinical samples, especially fecal, have been used for this investigation. Moreover, the frequency of the species may differ according to the region where they were isolated.

A study performed with diarrheal stool from 1,595 patients identified *Aeromonas* in 50 samples in India; the biochemical speciation was made on 35 strains showing that the most common species were *A. caviae* (34%), *A. veronii* biovar *veronii* (29%), *A. veronii* biovar *sobria* (26%), and *A. hydrophila* (9%; Mohan et al., 2017). In Kenya, 188 fecal samples were studied to determine the etiological agents of diarrhea, of which only five were caused by *A. hydrophila* and three by *A. caviae* (Mbuthia et al., 2018). In 16 hospitals in the city of Shanghai, China, 4,529 specimens from diarrheal patients were collected, in which 193 cases were related to *Aeromonas* infection: *A. veronii* (42.5%), *A. caviae* (25.3%), *A. aquariorum* (14.5%), *A. hydrophila* (5.7%), *A. enteropelogenes* (4.7%), *A. media* (3.1%), unknown (1.6%), *A. salmonicide* (2.1%), and *A. allosaccharophil*a (0.5%; Li et al., 2015). While a study at a hospital in Beijing city, China, 1,286 stool samples were analyzed from people with acute diarrhea, and 17 strains of *Aeromonas* causing extra-intestinal infections were identified in blood or bile. The distribution of *Aeromonas* species in these isolates obtained from intestinal and extra-intestinal samples followed *A. veronii* (31.3%), *A. caviae* (41.7%), *A. dhakensis* (13.9%), *A. media* (1.7%), *A. hydrophila* (5.2%), *A. sanarellii* (1.7%), *A. enteropelogenes* (1.7%), *A. bivalvium* (0.9%), and unknown (1.7%; Zhou et al., 2019).

During the period from January 2015 to December 2017 at Hospital Galdakao-Usansolo, Spain, 98 patients (having a median age of 62 years, and 51% of the cases were women) with positive stool cultures for *Aeromonas* were counted, being 85 cases of *A. caviae*, 12 of *A. veronii*, and 1 of *A. hydrophila*; estimating an occurrence of 32 cases for every 10^5^ inhabitants per year (Elorza et al., 2020).

Different biological samples, such as stool, eye, sputum, and blood, were tested in microbiological laboratories in Australia for *Aeromonas* search. From 100 isolates, 39 were *A. dhakensis*, 21 *A. veronii*, 20 *A. hydrophila*, 14 *A. caviae*, 4 *A. jandaei*, 1 *A. bestiarum*, and 1 *A. sanarellii* (Sinclair et al., 2016). A study of 109 *Aeromonas* clinical isolates from diarrhea patients was conducted in Mexico and Spain, and the most common species in both countries were *A. caviae*, *A. hydrophila*, and *A. veronii* (Aguilera-Arreola et al., 2007). A retrospective analysis from January 2006 to December 2012, carried out at the Hospital del Mar in Barcelona, Spain, detected 221 clinical samples positive for *Aeromonas* spp. in 204 patients. Gastroenteritis was the most common form of infection, comprising of 78.4% from patients. It was found that age above 80 years, admission to the intensive care unit, and malignancy were associated with increased mortality rate during the 1-year follow-up of infected patients (Nolla-Salas et al., 2017).

Regarding extra-intestinal infections caused by *Aeromonas*, there are varieties of sites that can be affected. A range of non-gastrointestinal infections can involve *Aeromonas* species, some have been associated with contaminated water, for example, soft tissue infection due to injury to an aquatic environment (Lujan-Hernandez et al., 2020), and wound infection after medical leech therapy that may have occurred due to contamination of the water where the leeches were bred (Masters et al., 2020).

*Aeromonas* wound or pus isolates were collected from a medical center in southern Taiwan, and 76 species were reported, *A. dhakensis* (37), *A. veronii* (14), *A. hydrophila* (13), *A. caviae* (11), and *A. media* (1; Chen et al., 2014b). Another study was conducted at this same medical center, in which among 514 stools from adults with diarrhea, 13 had *Aeromonas* detected, and of the 167 asymptomatic persons, only six were isolated. The most common species in these isolates were *A. veronii*, *A. caviae*, *A. sanarelli*, and *A. dhakensis* (Chen et al., 2014b). These data show that, although *Aeromonas* infections may occur in the same region, the difference in anatomical sites may reflect a distinct distribution among their isolate species.

## Pathological Processes and Clinical Manifestations

**Figure 1:**
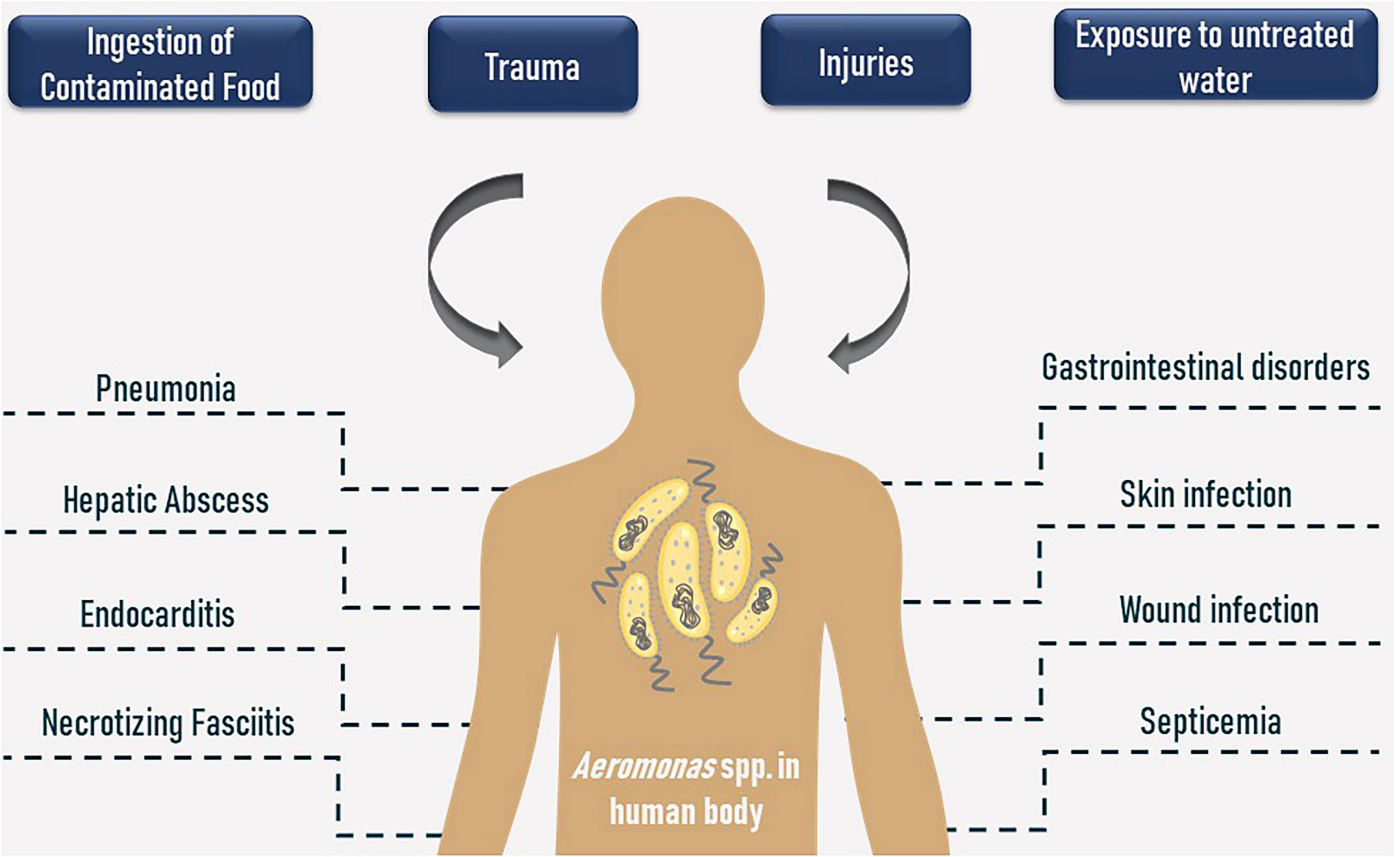
Contamination routes and human health disorders caused by *Aeromonas* spp.

Bacteria belonging to the *Aeromonas* genus are known as the main pathogens of fish and other marine animals, being responsible for deleterious outbreaks in aquaculture systems due to their wide distribution in various aquatic environments (Beaz-Hidalgo and Figueras, 2013; Liu, 2015; Figueras and Ashbolt, 2019). Moreover, they were already detected in bugs, reptiles, amphibians, birds, and other vertebrates (Percival and Williams, 2014; Praveen et al., 2016; Gonçalves Pessoa et al., 2019). Despite being firstly reported in 1891, the *Aeromonas* were only recognized as potential human pathogens about 60 years later, in 1954, when these microorganisms were detected in clinical samples obtained from an immunosuppressed woman who died due to a fulminant metastatic myositis (Parker and Shaw, 2011; Liu, 2015; Fernández-Bravo and Figueras, 2020). Currently, it is known that these bacteria can cause predominantly gastrointestinal tract disorders as well as infections in wounds, soft tissues, muscles, lungs, bones, and septicemia, to name a few (Parker and Shaw, 2011; Bhowmick and Bhattacharjee, 2018; Fernández-Bravo and Figueras, 2020). The main illnesses provoked by Aeromonads are shown in Figure 1.

*Aeromonas* species are classified into two main groups: (i) psychrophilic, usually non-motile strains which grow well between 22°C and 28°C, and (ii) mesophilic, motile with desirable growth temperature into 35°C–37°C or higher (Parker and Shaw, 2011; Percival and Williams, 2014; Liu, 2015; Fernández-Bravo and Figueras, 2020). The psychrophilic group is represented by the *Aeromonas salmonicida*, which is described as an important cold-water fish pathogen (Fernández-Bravo and Figueras, 2020; Pérez-García et al., 2021). However, this bacterium has already been detected from blood samples of an infected 20-year-old man diagnosed with endocarditis (Salehi et al., 2019). On the other hand, the mesophilic group is often related to both fish and human infections and is mainly represented by *A. hydrophila*, *A. caviae*, and *A. veronii*, but it embraces the other *Aeromonas*’s species excepting the psychrophilic *A. salmonicida* (Percival and Williams, 2014; Pérez-García et al., 2021).

Broadly described as opportunistic bacteria, pathological processes generated by these microorganisms are frequently reported in immunosuppressed patients. However, even healthy persons, from any age, can be potential hosts to illnesses caused by *Aeromonas* (Chuang et al., 2011; Silva et al., 2017). The wide range of diseases generated by Aeromonads is characterized as a joint action of several virulence factors, such as lipases, proteases, hemolysins, aerolysins, cytotoxins, and enterotoxins, that contribute both to its survival and environmental adaptation as well as to the multifactorial and complexity of its pathogenesis, whose mechanisms and processes are usually not evident (Pemberton et al., 1997; Zhou et al., 2013; Rasmussen-Ivey et al., 2016; Hoel et al., 2017).

Aforementioned, *Aeromonas* are natural habitants from aquatic environments and could be detected in samples obtained from different sources (Praveen et al., 2016; Harnisz and Korzeniewska, 2018). Furthermore, these bacteria can resist potable water treatment processes for human consumption, representing a potential contamination route (Janda and Abbott, 2010). Phylogenetic studies previously performed suggest feasible relations between *Aeromonas* strains isolated from both municipal water and clinical samples within the same geographic region (Pablos et al., 2009, 2011). The intake of contaminated food like animal-derived proteins (meat, chicken, and, mainly, fisheries), vegetables, and, additionally, the preparation methods of some culinary recipes are reported as the main cause for Aeromonads infections (Castro-Escarpulli et al., 2003; Pablos et al., 2009; Hoel et al., 2017). Environmental factors can influence outbreaks caused by these microorganisms. Studies have shown that temperatures over 22°C might provoke loss of virulence factors in *A. salmonicida* strains (Fernández-Bravo and Figueras, 2020). On the other hand, it has been reported that different temperatures modulate the expression of virulence genes in distinct isolates of *A. hydrophila*; most of the assessed genes were expressed by culturing the strains at 28°C (Pattanayak et al., 2020). Moreover, it was demonstrated that an *A. hydrophila* strain endured under low temperatures and starvation by shaping its virulence factors content (Bruscolini et al., 2014). Other *Aeromonas* species related to human health disorders grow well in higher temperatures and, consequently, lead to an increase in the number of infections under warm seasons due to the high bacterial cell count in the environment, especially in water (Bhowmick and Bhattacharjee, 2018).

Herein, based on case reports, the main clinical manifestations developed during infectious processes generated by *Aeromonas* will be discussed; reinforcing its relevance in public health and the importance of researches involving this bacterial genus in the field of medical microbiology.

### Gastrointestinal Diseases

Acute gastroenteritis is one of the most common diseases faced by physicians in emergency medical services around the world. It is presented as a sudden diarrheal process that may be followed by other symptoms like vomiting, abdominal pain, fever, nausea, and malaise, which are results from infectious processes, with inflammatory nature or not, involving the small intestine or the colon (Duman et al., 2015; Barrett and Fhogartaigh, 2017; Fleckenstein et al., 2021). In general, it is mainly caused by a viral infection, but several bacterial species have been associated with gastroenteritis episodes, representing from 15% to 40% of the reported cases, approximately (Barrett and Fhogartaigh, 2017; Schuetz, 2019).

The classification of *Aeromonas* as true enteropathogens has been under discussion for a long time. Among the main controversial arguments were (i) the lack of reliable identification of *Aeromonas* strains related to specific outbreaks, (ii) non-compliance with the criteria proposed in the Henle–Koch postulates, in regards to the isolation of the microorganism from the diseased body and reproduction of the infection using animal models, and (iii) lack of consistent evidence related to the induction of pathological processes in humans by these bacteria, which would confirm their pathogenicity (Janda and Abbott, 2010; Chen et al., 2015; Schuetz, 2019). Since previous outbreaks in which Aeromonads were detected on stool samples—obtained from infected patients—had already been reported, persisting on classifying these bacteria as true enteropathogens seem to be an obvious and logic conduct (Janda and Abbott, 2010). Such fact conflicted with the results of an experimental test involving the administration of oral solutions containing a high concentration of *Aeromonas* strains, where just two from a group of 57 tested humans experienced diarrhea, questioning the enteropathogenicity of these microorganisms (Morgan et al., 1985). However, according to different analysis of data obtained from different outbreaks involving Aeromonads and humans with diverse exposure profile, the potential of these bacteria on developing gastrointestinal disorders was finally proved. It was showed that the ingestion of low concentrations of these bacteria can develop infection even if asymptomatically; what attributed to the genus *Aeromonas* the classification of true enteropathogen, which severity of infections can be equally compared to those developed by other bacterial genus like *Campylobacter* and *Salmonella* (Teunis and Figueras, 2016).

Bacterial enteritis commonly originates from the ingestion of contaminated food, and microorganisms develop gastrointestinal disorders through the production of pre-toxins, adhesion to the epithelium, secretion of virulence factors, and the invasion of the intestinal mucosa, resulting in different symptoms (Barrett and Fhogartaigh, 2017; Fleckenstein et al., 2021). Several risk factors are associated, such as age group, immunological condition, and access to proper basic sanitation (Barrett and Fhogartaigh, 2017). When caused by *Aeromonas*, cases of gastroenteritis commonly present as episodes of self-limiting watery diarrhea, but they can also occur manifest more severely in the form of dysentery-like and cholera-like disease (Parker and Shaw, 2011; Harnisz and Korzeniewska, 2018). It has been reported a case of two women with a history of diarrhea, vomiting, and abdominal pain in a cholera endemic area. Although stool cultures and Gram staining indicated a possible infection by *Vibrio cholerae*, genomic analysis of the samples identified *A. caviae*, revealing Aeromonads enteritis mimicking cholera disease (Van Zwetselaar et al., 2018). Practically the same symptoms were presented by a 69-year-old patient who reported eating raw fish. Imaging examinations and physical evaluation revealed possible appendix perforation, but both the incompatibility of symptoms and detection of *A. hydrophila*/*caviae* by stool culture led to the final diagnosis of enteritis with evident mimicry of acute appendicitis (Kishimoto and Watari, 2018). Although considered common and usually simple to treat, gastroenteritis is one of the causes of bacterial gut translocation, which potentially leads to even more severe complications, such as peritonitis and colitis (Kim et al., 2018).

Peritonitis is an inflammatory process on the peritoneum, serous membrane that covers intra-abdominal organs, usually caused by bacterial infection (Lippi et al., 2014). In this case, it is classified mainly as primary, or spontaneous, and secondary. The former is a common complication of cirrhotic patients, contamination of the ascitic fluid (AF) as a result of overgrowing enteric bacteria and posterior translocation due to reduced motility of the intestine and low host defenses (Strauss and Caly, 2006). It was reported a case of a 57-year-old man, with a history of alcoholism and suffering from cirrhosis that came to death after developing septic shock from *A. hydrophila*, which was detected in blood cultures and ascetic fluid (Lin and Lin, 2019). In Taiwan, it was analyzed that, over 16 years, around 31 cases of cirrhotic patients developed spontaneous *Aeromonas* peritonitis. The main symptoms were fever and abdominal pain; these cases had a mortality rate of 56% (Wu et al., 2009). Secondary bacterial peritonitis is related to the presence of injuries or intestinal lesions caused basically by surgical procedures or trauma (Miyashita et al., 2019). This kind of infection has been reported in humans undergoing peritoneal dialysis (PD), a technique used in the management of patients suffering from end-stage renal disease (Gillis and Wilkie, 2019). Given that it is a home-based therapy, proper training of the patient for the correct handling of the device and also execution of fluid exchange is extremely important to avoid bacterial infections (Cho and Johnson, 2014; Gillis and Wilkie, 2019). A rare case of peritonitis related to PD due to *Aeromonas* infection has been reported. A 54-year-old man in the course of PD had washed a disposable part of the automated device with tap water and developed acute abdominal pain. Peritoneal dialysate culture detected *A. hydrophila*, and the patient was treated without device removal (Kim et al., 2018). Compared with the main Gram-negative bacteria that can cause peritonitis, *Aeromonas* cases are still considered uncommon, but not less lethal (Lippi et al., 2014).

Ulcerative colitis (UC), which compounds the group of inflammatory disorders involving the colon, does not have an exact pathogenesis (Cheng and Fischer, 2020). However, it is represented by an imbalance in the host’s immune system response to external antigens, such as food and commensal microorganisms (Cheng and Fischer, 2020; Du and Ha, 2020). A wide range of bacterial enteropathogens can develop infection through invading intestinal mucosa or secreting toxins that induce inflammation, causing tissue damage, such as erosions, ulcers, and even mimicking other chronic inflammatory bowel diseases, as a consequence of the persistence of the infectious process (Bhaijee et al., 2015). A case of a 34-year-old woman who developed abdominal pain and diarrhea about 3 days after eating fish was reported. The histopathological analysis detected inflammatory infiltrate and colon ulcers were found using endoscopy, which led to the diagnosis of UC. Molecular tests using fecal samples detected *Aeromonas* spp. (Morales-Fuentes et al., 2014).

### Skin, Soft Tissues and Wound Infections

Human skin consists of two layers that act as a physical barrier, protecting the body against external threats (Cefalu et al., 2017). Infections involving this extensive organ are one of the main disabling disorders in the world (Jabbour and Kanj, 2021). Characterized as invasions of the epidermis and adjacent tissues by virulent microorganisms, due to the presence of injuries and other risk factors, they initially generate common signs of local inflammation with a potential risk of evolution to various pathological processes, depending on the nature of the invading microbe (DiNubile and Lipsky, 2004; Dryden, 2010; Clebak and Malone, 2018). Naturally, the epidermis is colonized by several types of microorganisms and, when it comes to bacteria, the ones most commonly detected on the skin of adult individuals belong to the *Staphylococcus*, *Streptococcus*, and *Corynebacterium* genera (Cefalu et al., 2017; Byrd et al., 2018; Vasagar et al., 2018). Among the main bacterial skin infections are impetigo, erysipelas, cellulitis, folliculitis, abscesses, and necrotizing fasciitis (Clebak and Malone, 2018). Noteworthy, when such diseases are contracted through prior exposure to aquatic environments, the polymicrobial etiology of the infection must be taken into account, especially with regards to the presence of unusual microorganisms, such as *Aeromonas* (Vasagar et al., 2018).

Aeromonads potentially generates a variety of skin and soft tissue disorders, most often affecting healthy people, aging ≥10 years, who have suffered some type of injury (such as burns and trauma) and/or exposure to contaminated water (Janda and Abbott, 2010; Parker and Shaw, 2011; Fernández-Bravo and Figueras, 2020). *Aeromonas* species have been detected by the pus culture of infected wounds contained in a 12-year-old boy that had injured his knees after falling on rocks while swinging over water (Rutteman, 2017). Likewise, *A. hydrophila* and *E. cancerogenus* were also identified in the pus culture of an elderly patient who suffered a traffic accident and fell into a pool of contaminated water (Hadano et al., 2017). Traumatic events involving sharp objects have also been the cause of infections by these bacteria (Larka et al., 2003). Burn wounds have a high risk of contamination when not treated properly, mainly because they are exposed to water as a first-aid measure (Ribeiro et al., 2010). Although uncommon, some *Aeromonas* spp. have been reported as the cause of infection and worsening prognosis of patients in this situation (Kienzle et al., 2000; Chim and Song, 2007; Lai et al., 2007).

Although the vast majority of skin infections involving *Aeromonas* have been reported as a consequence of a previously caused injury, objects that are eventually filled with contaminated water and used for domestic or recreational purposes can also be a source of infection. Cases of *Aeromonas* folliculitis have been reported in children after playing in inflatable swimming pools filled with tap water without any purification or disinfection system. One of them, the 11-year-old girl, developed symptoms related to systemic inflammatory syndrome due to a left sinus infection that was not completely resolved until *Aeromonas* was detected in the purulent fluid collected (Olszewski et al., 2017). The other kids, a 15-year-old girl and an 8-year-old boy, complained of just pruritus due to the presence of multiple follicular lesions. Pustular fluid cultures detected *A. hydrophila* in the two patients, who used the inflatable pool for 5 days without water change (Manresa et al., 2009). Severely, a 34-year-old man developed folliculitis in the pubic region potentially caused by Aeromonads that resulted in rashes, swelling, and local alopecia. Since her partner had also developed similar symptoms and both denied extramarital relationships and sexually transmitted diseases, the possible cause of the infection was related to the use of a spa bath with no regular maintenance (Mulholland and Yong-Gee, 2008).

Regarding musculoskeletal and soft tissue infections, the *Aeromonas* are recognized to generate disorders in both healthy and immunocompromised patients (Voss et al., 1992; Janda and Abbott, 2010). Necrotizing fasciitis (NF), a disease associated with a high mortality rate, which usually needs surgical intervention, is characterized by a progressive infection that starts in the fascia and extends to the subcutaneous tissues, impairing local blood circulation, generating deep necrosis (Ali and Lateef, 2016; Narayan and McCoubrey, 2019; Stevens et al., 2021). NF is subdivided into four categories, depending on the type and number of microorganisms detected, and has several etiologies, risk factors, and pathogenic mechanisms (Narayan and McCoubrey, 2019; Stevens et al., 2021). Some *Aeromonas* species have been identified causing NF. A case involving an 8-year-old boy who injured his right foot while swimming and developed fever, as well as a general malaise in addition to progressive pain and edema in the affected region, was reported. During surgery, fascial necrosis was detected, and cultures of collected samples identified the presence of *A. hydrophila* and *S. pyogenes* (Fletscher Covaleda et al., 2013). On the other hand, the 80-year-old man with no previous trauma or exposure to contaminated water died from NF caused by Aeromonads, which led to multiple organ dysfunctions (Cui et al., 2007). *Aeromonas hydrophila*, *A. caviae*, and *A. dhakensis* were distinctly found causing fatal NF in immunocompromised patients suffering from a wide range of underlying diseases like heart failure, hypertension, leukemia, aplastic anemia, cirrhosis, and diabetes (Spadaro et al., 2014; Hong et al., 2018; Ugarte-Torres et al., 2018) as well as acting as secondary pathogens in dengue patients (Chang et al., 2018). *Aeromonas caviae* was also detected in a case of a 22-year-old woman that developed NF after an aesthetic surgical procedure in both calves (Park et al., 2010).

### *Aeromonas* Bacteremia and Sepsis

When searching through the literature, it is easy to identify differences in the application of the expressions “bacteremia” and “sepsis” concerning systemic infections caused by bacteria and other microorganisms (Søgaard et al., 2012). Bacteremia refers to the presence of bacteria in the bloodstream, while sepsis is characterized as a systemic inflammatory syndrome involving components of the innate immune system, which can be caused by bacterial pathogens as well as fungi, viruses, and parasites; resulting in other metabolic disorders which lead to multiple organ failure and patient death (Girard and Ely, 2007; Perner et al., 2016; Minasyan, 2019). *Aeromonas* are recognized in the group of Gram-negative bacteria causing bacteremia/sepsis, but they do not cause any signs or symptoms that distinguishes their systemic infections from cases involving other bacterial genera (Janda and Abbott, 2010). The incidence of Aeromonas septicemia is relatively low. Its mortality rate can vary between 25% and 30% and it is commonly related to immunocompromised patients or those suffering from underlying diseases (Parker and Shaw, 2011; Fernández-Bravo and Figueras, 2020). *Aeromonas hydrophila* was reported in a rare case of fulminant septicemia in a child in the course of acute lymphoblastic leukemia therapy, generating tissue necrosis and requiring extensive surgical debridement (Papadakis et al., 2012). *Aeromonas sobria* was the cause of fatal septicemia in a patient living with HIV. The infection developed rapidly, the patient’s health condition has severely deteriorated and the pathogen was discovered in post-mortem tests (Stano et al., 2009). In cases of sepsis, rapid diagnosis and the initiation of appropriate empirical antibiotic therapy have been cited as crucial measures to preserve the life of the affected patient (Minasyan, 2019; Sweeney et al., 2019).

### Hepatobiliary and Pancreatic System Infections

Infectious diseases affecting the liver and the biliary tract trigger the development of other complications of human health, which demand rapid and accurate diagnosis (Hynes et al., 2020). The potential of *Aeromonas* in generating disorders in these systems has already been described, with cases of acute suppurative cholangitis (ASC), an obstructive and infectious complication of the biliary tract, being one of the most common reports (Janda and Abbott, 2010; Sulzer and Ocuin, 2019). In a general evaluation of patients diagnosed with ASC, symptoms, such as fever, jaundice, abdominal pain, and the presence of several underlying conditions, were verified. *Aeromonas hydrophila* was the most detected species in bile cultures, *A. caviae* and *A. veronii* biotype *sobria* were also found (Chan et al., 2000). In another analysis, the same species were also isolated from bile samples collected from 750 patients suffering from biliary tract infections. The authors highlighted the tendency of cases to occur frequently in patients with immunosuppressive conditions due to diseases, such as cancer, diabetes, cirrhosis, and other liver illnesses, in addition to therapy with immunosuppressive agents (Chao et al., 2013b). Invasive clinical procedures also pose a risk of bile system infections by *Aeromonas*. A case of a 64-year-old woman who developed sepsis and other complications from *A. veronii* biovar *veronii* after biliary drainage catheter insertion was reported (Monti et al., 2019). *Aeromonas* were also the cause of liver and pancreas abscesses (Kratzke and Golenbock, 1987; De Gascun et al., 2007).

### Other Infections

Bacteria belonging to the genus *Aeromonas* have been uncommonly reported generating disorders in several other organs, such as eyes, lungs, bones, and kidneys, for example, (Bhowmick and Bhattacharjee, 2018; Fernández-Bravo and Figueras, 2020).

Regarding eye infections, a case of a 35-year-old man who developed keratitis related to the use of contact lenses, which was occasionally exposed to tap water, was reported. *A. caviae* was detected by culturing eye swabs and contact lens case (Pinna et al., 2004). Aeromonads were also the cause of conjunctivitis, contracted in a nosocomial route, in a diabetic patient (Gonzales et al., 1989). Such disorders can also occur as a consequence of other previously developed diseases. It was reported a case of a 79-year-old woman who developed endogenous endophthalmitis, eye infection involving the vitreous and/or aqueous humor, after developing *Aeromonas*-related gastroenteritis with progression to sepsis (Durand, 2013; Ryan et al., 2017).

The species *A. veronii* and *A. hydrophila* have been related to cases of pneumonia in both immunocompromised and immunocompetent patients with or without prior contact with suspicious water samples (David Reines and Cook, 1981; Ku and Yu, 2017). Such complication was also reported as a consequence of a near-drowning case of a 43-year-old man, whose clinical course evolved to multiple organ failure and death (Ender et al., 1996).

Cases involving genitourinary tract infections by *Aeromonas* species have been generally described as infrequent episodes, usually reported in patients with reduced immunity or undergoing invasive therapeutical procedures (Chao et al., 2012). *Aeromonas hydrophila* was found to be the cause of hematuria in a 42-year-old patient with a history of kidney transplantation (Hussain et al., 2018). The same species was isolated by peripheral veins and dialysis catheter cultures of a 55-year-old man that experienced kidney failure and was initiated on hemodialysis (Khalil et al., 2013). The insertion of these medical devices has been related to a large percentage of cases of bloodstream infections, and the treatment duration has been one of the risk factors for contamination by microorganisms (Kumbar and Yee, 2019). It was reported a case of a 42-year-old woman who died of bacteremia involving *A. hydrophila* in the course of hemodialysis sessions due to chronic kidney failure (Lin et al., 1996). *Aeromonas caviae* and *A. veronii* have also been detected as the cause of genitourinary tract infections in both healthy and immunosuppressed patients (Chao et al., 2012).

The involvement of the bones and joints due to infections represents a serious health problem, which can mainly culminate in the patient’s disability (Bejon, 2017). The potential of Aeromonads to colonize and infect these organs has already been reported, especially as a consequence of a previous wound or exposure trauma contamination (Janda and Abbott, 2010). *Aeromonas sobria* was the cause of ethmoiditis in a 16-year-old boy who experienced fever, eye swelling, vomiting, and headache after playing in a river (Couturier et al., 2017). *Aeromonas hydrophila* was detected from blood cultures of a cirrhotic patient who developed bacteremia followed by acute osteomyelitis (Lee et al., 2003). Similarly, the same species was found to be the cause of chronic osteomyelitis in a 50-year-old diabetic patient (Agrawal et al., 2017).

## Differential Diagnosis

Although *Aeromonas* species can cause extraintestinal infections, such as skin and soft-tissue infections, one of the main consequences of *Aeromonas* infection is gastroenteritis, as observed according to the epidemiological data presented. Microbiology laboratories do not usually investigate *Aeromonas* spp. in diarrheal stools. The most commonly searched bacteria are *Salmonella*, *Shigella*, *Campylobacter*, and *Escherichia coli* (Schuetz, 2019).

Gastroenteritis may be caused by viral, bacterial, and parasitic pathogens whose clinical symptoms may be similar, so analysis with the patients’ stool becomes indispensable to promote differential diagnosis. *Aeromonas* spp. can grow in routine culture media commonly used in clinical laboratories, such as sheep blood agar (SBA) and chocolate agar. In addition, these microorganisms also grow in specific culture media for the isolation of enteropathogenic bacteria, for example, hektoen enteric agar, xylose deoxycholate agar (XLD) agar, and MacConkey agar (Carr, 2016). Moreover, *Aeromonas* recovery has been facilitated using enrichment broths, for example, alkaline peptone water under overnight incubation and subcultured onto blood ampicillin and cefsulodin irgasan novobiocin (CIN) agars (Igbinosa et al., 2012).

In addition to growth in culture medium, it is important to perform other phenotypic tests, such as different biochemical tests, for more accurate identification. For example, the fact that most *Aeromonas* organisms have a positive oxidase reaction, resistance to the vibriostatic compound O/129, absence of ornithine decarboxylase activity, and no growth in 6% NaCl allows their differentiation with the genera *Vibrio* and *Plesiomonas* (Batra et al., 2016). *Aeromonas* can grow on selective and differential enteric agars; however, the carbohydrate contained in these agars can influence the growth of these microorganisms and is therefore not ideal for primary isolation in fecal samples of *Aeromonas*. For example, the ability to ferment carbohydrates can depend on the species, carbohydrates, such as xylose and lactose, can inhibit the growth of some *Aeromonas*, and a false-negative oxidase reaction can occur due to an acidification process in the medium (Oliveira et al., 2020).

However, the laboratory screening using phenotypic tests can still generate doubts about the correct diagnosis of the bacteria causing the infection; for example, the distinction between *V. cholera* and *Aeromonas* spp. in stool samples from patients with diarrheal episodes can only be achieved with genome sequencing analysis (Van Zwetselaar et al., 2018). The molecular identification of *Aeromonas* species can be performed through 16S rRNA gene analysis, housekeeping genes, genotyping techniques, the latter includes different methods, such as multilocus sequence typing (MLST) and enterobacterial repetitive intergenic consensus-PCR (ERIC-PCR; Fernández-Bravo and Figueras, 2020). *Aeromonas* specification is not commonly performed clinically, although its species can be identified quickly, for example by employing matrix-assisted laser desorption–ionization time-of-flight mass spectrometry (MALDI-TOF; Benagli et al., 2012; Akyar and Can, 2013; Chen et al., 2014a).

## Antibiotic Responses and Therapeutic Efficacy

Prescribing antibiotics in clinical practice was undoubtedly a revolutionary milestone in medicine regarding therapy against bacterial infections (Baron et al., 2017). The antibiotics era, which began around 1930 until the current days, has saved countless lives throughout the story. However, it brought one of the major and unavoidable public health problems of the modern world: the generation of multi-resistant bacteria (Dimopoulos et al., 2016; Baron et al., 2017; Dodds, 2017; Sánchez and Demain, 2017). The uncontrolled usage and irregular disposal of antibiotics, among other chemical compounds, that is, several drugs and personal use products, promote a propitious environment for the development of hard-to-treat microorganisms that become a threat to human health by sharing and acquiring resistance mechanisms which they naturally might not have (Chaturvedi et al., 2021). Although there has been great concern in recent years about environmental contamination by chemical and antimicrobial compounds, with a consequent increase in resistant microorganisms (Figueira et al., 2011), the history of humanity’s relationship with antibiotics has established a cycle of dependence that—apparently—will no sooner end. These medicines are still our front line in the fight against bacterial infections, and their consumption is estimated to globally increase approximately 200% by 2030 (Klein et al., 2018; Zheng et al., 2021).

Wastewater effluents derived from hospitals, veterinary clinics, industries, and aquaculture farms make aquatic environments great spreaders of multi-resistant pathogens (Baquero et al., 2008; Baron et al., 2017). Thus, *Aeromonas*, as natural inhabitants of these ecosystems, have a wide antibiotic resistance profile and are constantly isolated from several species of fish and other animals (Beaz-Hidalgo and Figueras, 2013). Aeromonads acquire and share antibiotic resistance genes by transmitting mobile elements like plasmids, integrons, insertion sequences, and transposons (Piotrowska and Popowska, 2015; Stratev and Odeyemi, 2016). These genetic compounds are known as the “mobilome” and are permanently subjected to evolution according to changes in the environment (Piotrowska and Popowska, 2015).

It has been described the production of 4 main groups of beta-lactamases, divided from Class A to Class D, which confer to the *Aeromonas* a potential defense against the action of beta-lactam drugs (Stratev and Odeyemi, 2016; Fernández-Bravo and Figueras, 2020). The antimicrobial resistance of 24 strains of *Aeromonas* isolated from Nile tilapia and domestic fowl was evaluated. The strains showed to be invulnerable to the beta-lactams amoxicillin and ampicillin/sulbactam, as well as to streptomycin, from aminoglycoside’s class (Abu-Elala et al., 2015). Aeromonads isolates from cow fecal samples from different farms also showed complete resistance to a large group of beta-lactam antibiotics and susceptibility to cephamycin, tetracycline, ciprofloxacin, and gentamicin (Igbinosa et al., 2015). In addition, sensitivity to cefepime was also observed in strains obtained from commercialized/farmed shellfish as well as aquatic environment samples (Chen et al., 2021). Aeromonads from cultured freshwater fishes showed main resistance to ampicillin and amoxicillin and absolute sensitivity to levofloxacin (Azzam-Sayuti et al., 2021). Besides beta-lactams, resistance against colistin can also be chromosomally mediated or transferred by the mobilome. This antibiotic can disrupt the membrane of gram-negative bacteria by interacting with surface LPS and is used as last resort for infections cause by multi-drug resistant microorganisms (MDR). Reports showed the existence of colistin-resistant Aeromonads, especially among the isolates obtained from clinical samples (Gonzalez-Avila et al., 2021).

*Aeromonas* are also capable of colonizing water treatment plants and, consequently, contaminate both the content intended for human consumption and the environment in which effluents are disposed (Figueira et al., 2011). Strains obtained from a domestic and hospital treatment system have been shown to resist three or more classes of antibiotics, including tigecycline, another drug used as a last line in cases involving MDR (Harnisz and Korzeniewska, 2018). The hazardousness of the dissemination of these microorganisms in the environment, arising from the addition of hospital waste to domestic effluents in municipal water treatment systems, has already been reported (Varela et al., 2016).

Although the wide antibiotic resistance and susceptibility profiles within the *Aeromonas* genus, it is believed that the therapeutic administration of these drugs should not be restricted to specific species (Janda and Abbott, 2010). Strains of *A. trota* obtained from clinical samples were susceptible to ampicillin, an antibiotic generally not recommended for the treatment of infections caused by this genus (Carnahan et al., 1991; Janda and Abbott, 2010; Bhowmick and Bhattacharjee, 2018). In addition, (Hughes et al., 2016) isolated *A. veronii* from a stool culture and found that this strain was resistant to ertapenem and susceptible to imipenem and meropenem. These same authors also identified in perirectal isolates from two different patients the species *A. veronii* and *A. hydrophila*, which were resistant to all carbapenems previously cited. Carbapenems are widely used for therapy against MDR infections and resistance to this antibiotic is a global concern (Nordmann and Poirel, 2019; Kaluba et al., 2021). Previous detection of carbapenem-resistant bacteria is extremely important to improve the patient’s prognosis and thus reduce mortality. Noteworthy, the execution of the antibiogram test in clinical practice is crucial in prescribing antibiotics able to contribute to the therapeutic process (Hughes et al., 2016; Nordmann and Poirel, 2019).

Symptoms of acute gastroenteritis present commonly as self-limiting within approximately 5 days (Graves, 2013). Dehydration, caused by successive diarrheal episodes, is reversed by oral or intravenous rehydration techniques to balance body fluids and electrolytes (Graves, 2013; Mosegui et al., 2019). In these cases, antibiotics have been prescribed to quickly reduce the frequency of diarrhea, shorten the recovery time, and control the contagion, since it reduces the release of the pathogen in stools (Graves, 2013; Cohen et al., 2017). When dealing with antibiotics, the appropriate choice of empirical treatment before antibiogram results is very important, especially for reducing bacterial resistance (Zhu et al., 2021). *Aeromonas* spp. isolates from stool samples from children in a diarrheal outbreak demonstrated susceptibility to gentamicin, amicin, and cefepime (Soltan Dallal et al., 2016). Ciprofloxacin was used empirically in association with rehydration therapy in cases of gastroenteritis by *A. hydrophila* (Van Zwetselaar et al., 2018). Oral prescription of the same antibiotic was reported according to the results of the antibiogram performed with an *A. hydrophila* strain detected by stool culture from a patient experiencing gastroenteritis (Ahishali et al., 2007). Empirical infusion of sulbactam/ampicillin was used to treat enteritis by *Aeromonas*, with subsequent change of therapy by oral levofloxacin (Kishimoto and Watari, 2018).

Considering the therapy against peritonitis associated with PD, it is recommended by the International Society for Peritoneal Dialysis that antibiotics should be prescribed even if the infection is only a suspect; being administrated preferably by intraperitoneal route (Al Sahlawi et al., 2020). Vancomycin and ceftazidime were used empirically in a case of peritonitis involving a PD patient. Based on susceptibility testing, only ceftazidime was continued in the course of treatment of *A. hydrophila* infection (Kim et al., 2018). In a similar case involving *A. sobria*, the PD patient was treated with empirical teicoplanin and cefotiam. According to the antibiogram, therapy was replaced by intraperitoneal and intravenous infusion of amikacin and levofloxacin, respectively (Song et al., 2019).

Skin infections are classified according to the location of the lesion, analysis of its extension, and degree of involvement of the adjacent superficial and deep tissues (Ramakrishnan et al., 2015; Jabbour and Kanj, 2021). Thus, the therapeutic strategy can range from topical treatment to administrate antibiotics and surgical procedures to remove necrotic tissues (Jabbour and Kanj, 2021). Due to the multiple causes and origins of lesions and skin contamination, emergencies usually require rapid administration of empirical antibiotics, before susceptibility testing, to preserve the patient’s life and eventually reduce the need for invasive approaches (Abrahamian et al., 2008). *Aeromonas hydrophila* has been frequently reported in several cases of skin infection with previous trauma. Gentamicin and carbenicillin were used empirically to treat an infection in a puncture wound. Culture of purulent material and subsequent susceptibility testing isolated a strain of *A. hydrophila* sensitive to the aminoglycoside class, tetracycline, and chloramphenicol (Katz and Smith, 1980). The same species was detected in a case of postoperative sepsis due to a hand injury. Antibiogram revealed strain sensitivity to different classes of antibiotics, but resistance to amoxicillin/clavulanic acid, erythromycin, and clindamycin (Yang et al., 2004). *Aeromonas hydrophila* was also detected in purulent samples collected during surgical incision at sites of foot trauma infection. Levofloxacin and ampicillin/sulbactam were used empirically for adult and pediatric patients, respectively. After test results, the strains obtained from the cultures demonstrated susceptibility to levofloxacin and trimethoprim/sulfamethoxazole (Larka et al., 2003). *Aeromonas* species were detected in a study involving 129 cancer patients who experienced polymicrobial skin and soft tissue infections without previous exposure to water. Therapeutic administration of ceftazidime, ciprofloxacin, and ceftriaxone was reported (Chao et al., 2013a).

The management of other skin and soft tissue infections has also been reported. Aforementioned, *A. hydrophila* has been the underlying cause of folliculitis (Mulholland and Yong-Gee, 2008; Manresa et al., 2009; Olszewski et al., 2017). In these cases, the main therapeutical strategy has been using topical drugs and oral antibiotics have been chosen as a second-line treatment if there is no improvement in the infectious scenario (Clebak and Malone, 2018). Topical administration of gentamicin was prescribed to treat pediatric patients experiencing *Aeromonas* folliculitis (Manresa et al., 2009). The isolated strain of *A. hydrophila* has also been shown to be susceptible to other antibiotics. In the case of spa bath folliculitis, oral dicloxacillin was used empirically, being later replaced by oral ciprofloxacin according to antibiogram (Mulholland and Yong-Gee, 2008). Empirical use of ampicillin/sulbactam did not contain the progression of infection in a patient suffering from NF by *A. sobria*. Therapy was initially changed to ceftriaxone and levofloxacin and then to meropenem and linezolid. Susceptibility testing revealed resistance of the strain to meropenem, which was discontinued and replaced by ceftazidime (Spadaro et al., 2014). On the other hand, the prescription of meropenem was crucial in the prognosis of a patient diagnosed with NF by *A. caviae*, whose infection was also not controlled by empirical administration of antibiotics (Park et al., 2010). In these cases, in addition to drug therapy, surgical procedures of debridement and fasciotomies are also critical in the treatment and survival of the affected patient (Clebak and Malone, 2018). Other therapeutical strategies for uncommon *Aeromonas* human infections are summarized in Table 1.

**Table 1:** Antimicrobial strategies and respective outcomes for uncommon *Aeromonas* human health disorders.

Disease	*Aeromonas* species	Empirical antibiotics	Therapeutical antibiotics	Therapeutical antibiotics dosage	Treatment length	Outcome	References
Brain abscess	*A. hydrophila*	Ceftriaxone and vancomycin	Ceftriaxone	NM	NM	Death	Mahabeer et al., 2014
Endocarditis	*A. salmonicida*	NM^*^	Ceftriaxone and cefixime	1 g twice a day (hospital) and 400 mg daily (home), respectively	4 weeks (hospital) and 4 weeks (home)	Cure	Salehi et al., 2019
Endophthalmitis	*Aeromonas* spp.	Amoxicillin/Clavulanate piperacillin/Tazobactam ciprofloxacin	Ceftriaxone and ciprofloxacin	NM	NM	Death	Ryan et al., 2017
Ethmoiditis	*A. sobria*	Cefotaxime and fosfomycin	Ciprofloxacin	500 mg twice a day	3 weeks	Cure	Couturier et al., 2017
Keratitis	*A. caviae*	NM	Tobramycin, ciprofloxacin, and homatropine	1.5% hourly; 0.3% 6 × day and 1% 2 × day, respectively	10 days	Cure	Pinna et al., 2004
Osteomyelitis	*A. hydrophila*	Amoxicillin/Clavulanic acid	Ciprofloxacin	500 mg 12 hourly	6 weeks	Cure	Agrawal et al., 2017
Pancreatitis	*A. hydrophila*	Piperacillin/Tazobactam	NM	NM	NM	Death	De Gascun et al., 2007
Pneumonia	*A. veronii*	Piperacillin/Tazobactam and minocycline	Meropenem and levofloxacin	1 g 8 hourly and 750 mg daily, respectively	4 weeks	Death	Ku and Yu, 2017
Pyomyositis	*A. hydrophila*	Cefoxitin	Gentamicin and trimethoprim/Sulfamethoxazole	NM	6 weeks	Cure	Kratzke and Golenbock, 1987
Septic arthritis	*A. hydrophila*	Vancomycin and ceftazidime	Ciprofloxacin	750 mg 12 hourly	4 weeks	Cure	Fernández-Serna et al., 2013

* *NM, not mentioned.*

## Conclusion

For a long time, the genus *Aeromonas* was erroneously dissociated from the group of bacterial pathogens that have caused infections in humans. Some signs, like several diarrheal outbreaks around the world and widespread distribution of the *Aeromonas* in the environment reveal that these microorganisms were present in the medical routine for a very long time. Moreover, a great number of lives were taken under the consequences of its infectious processes. Given the diversity of clinical manifestations that Aeromonads can cause in both immunocompromised and immunocompetent patients, physicians should not underestimate this bacterial genus. As emergent pathogens, *Aeromonas* species are as common as other bacterial pathogens and are more prone to acquire multi-drug resistance, representing a severe threat to human health in the future. Therefore, progress in scientific studies aiming at new methods for *Aeromonas* identification as well as appropriate antimicrobial strategies will be a potential help for clinical approaches.

## Author Contributions

RBGP and LCBBC designed the structure of the manuscript. RBGP and WFdO wrote the review. MTdSC, AF, and LCBBC reviewed and approved the final version of the manuscript. All authors contributed to the article and approved the submitted version.

## Funding

Conselho Nacional de Desenvolvimento Científico e Tecnológico, Fundação de Amparo à Ciência e Tecnologia do Estado de Pernambuco, and Coordenação de Aperfeiçoamento de Pessoal de Nível Superior were responsible for fellowship.

## Conflict of Interest

The authors declare that the research was conducted in the absence of any commercial or financial relationships that could be construed as a potential conflict of interest.

## Publisher’s Note

All claims expressed in this article are solely those of the authors and do not necessarily represent those of their affiliated organizations, or those of the publisher, the editors and the reviewers. Any product that may be evaluated in this article, or claim that may be made by its manufacturer, is not guaranteed or endorsed by the publisher.
